# Traumatic Ovarian Artery Pseudoaneurysm Post-ovum Pickup: A Rare Complication

**DOI:** 10.7759/cureus.20825

**Published:** 2021-12-30

**Authors:** Afnan F Almuhanna, Maryam A Alkhalifa, Fatimah Y Altaweel, Kawther S Altaweel, Roaya A Al Qunais, Kawthar M Alsawad

**Affiliations:** 1 Radiology, King Fahad University Hospital, Khobar, SAU; 2 Radiology, College of Medicine, Imam Abdulrahman Bin Faisal University, Dammam, SAU

**Keywords:** pseudoaneurysm, case report, traumatic, embolization, aneurysm

## Abstract

A uterine/ovarian artery pseudoaneurysm is a rare complication of pelvic surgical interventions such as oocyte retrieval. This complication can be life-threatening due to the risk of rupture and blood extravasation. We report the case of a 30-year-old, nulliparous, married woman with a rare uterine/ovarian artery pseudoaneurysm due to in vitro fertilization. The patient has a history of sickle cell disease and primary infertility with multiple failed ovulation induction. She presented to the emergency department (ED) with hypotension following ovum retrieval for in vitro fertilization. Upon examination, we noted that the patient had tense ascites. A focused assessment with sonography in trauma (FAST) in the ED revealed internal bleeding. The diagnosis of a uterine artery pseudoaneurysm was established. She underwent an exploratory laparotomy to control bleeding due to her instability. Computed tomography and FAST scans help make an accurate diagnosis, and this case highlights that early intervention by embolization is essential to stabilize the patient and improve patient outcomes.

## Introduction

Pelvic surgical interventions, such as oocyte retrieval, carry a risk of complications, including the rare complication of a uterine/ovarian artery pseudoaneurysm, which can be life-threatening given the chance of rupture and blood extravasation [1]. Transvaginal oocyte retrieval is an ultrasound-guided technique in which oocytes are aspirated using a needle connected to a suction pump. Clinical complications after transvaginal oocyte retrieval include bleeding, infection, urinary tract injury, and pseudoaneurysm. In a ruptured aneurysm, bleeding can last 24 hours to six weeks [1-2]. We report a case of traumatic ovarian pseudoaneurysm following oocyte retrieval in a 30-year-old woman. In such a case, urgent management is required, which includes stabilizing the patient hemodynamically and then intervening in the ruptured artery by embolization [3].

## Case presentation

A 30-year-old, nulliparous, married woman presented to the emergency department (ED) with hypotension post-ovum retrieval for in vitro fertilization (IVF) followed by clinical deterioration and hypovolemic shock with internal bleeding. She had a history of sickle cell disease and primary infertility with multiple failed ovulation induction, the last of which was on the day of presentation. Her body temperature was 36.2°C, pulse was 109 beats per minute, respiratory rate was 17 breaths per minute, blood pressure was 89/55 mmHg, and oxygen saturation was 99% on room air. An abdominal examination revealed tense ascites. Table 1 presents the patient’s laboratory investigations.

**Table 1:** Laboratory investigations upon admission Abbreviation: PT/INR, prothrombin time/international normalized ratio

Laboratory test	Patient results	Reference range
White blood count	8.9 k/µl	4-11 k/µl
Red blood count	0.96 Mil/µl	4.2-55 Mil/µl
Hemoglobin	2.9 g/dL	12-16 g/dL
Hematocrit	8.5%	37-47%
Mean corpuscular volume	89.3 fL	80-94 fL
Platelets	34 k/µl	140-450 k/µl
PT/INR	22.4 seconds	12.9-15.9 seconds
Activated partial prothrombin time	49.2 seconds	25-42.3 seconds

**Figure 1:**
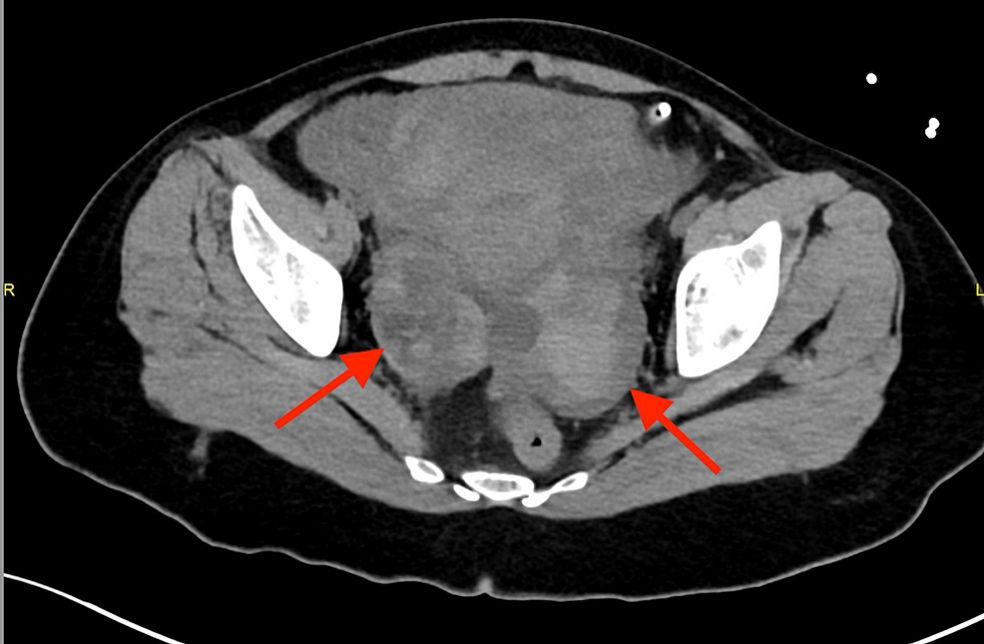
Enlarged ovaries with multiple hyperdense follicles and hyperdense free fluids

**Figure 2:**
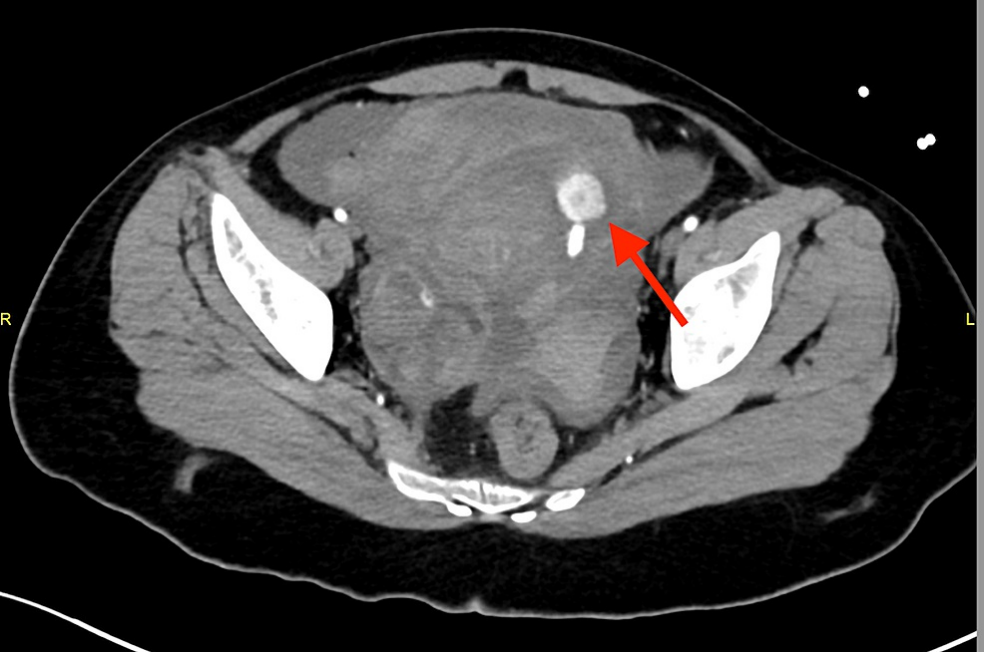
Extravasation of contrast denoting active bleed

**Figure 3:**
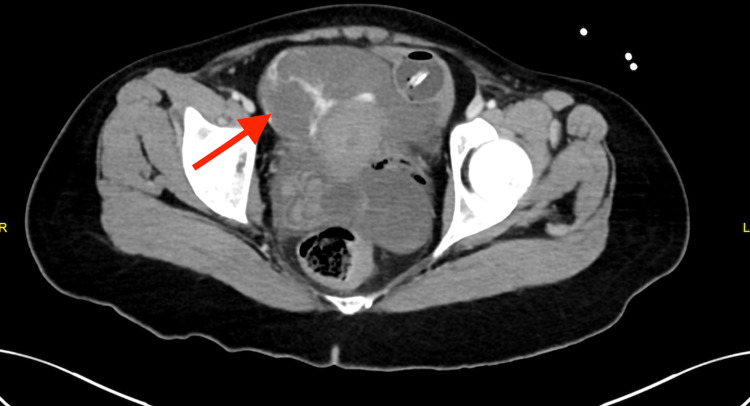
Extravasation of contrast denoting active bleed

In the ED, a focused assessment with sonography in trauma (FAST) scan revealed internal bleeding. A computed tomography (CT) scan revealed active extravasation adjacent to the hyperstimulated left ovary and severe hemoperitoneum (Figures 1-3).

The patient underwent an emergency exploratory laparotomy. We noted a massive hemoperitoneum reaching the liver with bilateral active oozing points in both ovaries, then applied surgical ligation to maintain hemostasis. Additionally, the patient received five units of packed red blood cells and six units of fresh frozen plasma. Table 2 presents her laboratory results following the blood transfusion.

**Table 2:** Laboratory investigation after blood transfusion Abbreviation: PT/INR, prothrombin time/international normalized ratio

Laboratory test	Patient Results	Reference Range
White blood count	15.4 k/µl	4-11 k/µl
Red blood count	4.38 Mil/µl	4.2-55 Mil/µl
Hemoglobin	11.9 g/dL	12-16 g/dL
Hematocrit	34.4%	37-47%
Mean corpuscular volume	78.6 fL	80-94 fL
Platelets	59 k/µl	140-450 k/µl
PT/INR	15.4 seconds	12.9-15.9 seconds
Activated partial prothrombin time	33 seconds	25-42.3 seconds

We measured her complete blood count every three hours to assess her hemoglobin response post-transfusion. Following the intervention, she shifted to the surgical intensive care unit where she was intubated, her blood pressure was maintained, and she kept on broad-spectrum antibiotics. Four days following the procedure, she returned to the regular ward and was vitally stable and tolerating a regular diet. The patient was evaluated daily clinically and with laboratory investigations. On postoperative day nine, she was vitally stable, her hemoglobin was 13.7 g/dL, and her abdomen was soft, lax, and nontender. Her surgical scars were well-healed, and she was discharged home with instructions to follow up.

## Discussion

Primary infertility is recognized as a serious, costly, and challenging health problem that impacts patients individually and collectively in society [4]. Primary infertility is defined as the inability of couples to conceive after 12 months of regular unprotected intercourse. A literature review in Tehran showed that the youngest age at marriage is associated with the highest infertility rate [5]. Infertility affects nearly 8% to 10% of couples worldwide. The World Health Organization reported that in low-income countries, one in four couples has infertility. Worldwide, 60 to 80 million couples are affected by infertility each year [4-5]. In 2012, a study in Saudi Arabia reported that of 2,414 patients who attended the obstetrics and gynecology clinic, 15.24% had primary infertility [6]. Given its magnitude, the problem desperately needs urgent action, mainly because most infertility cases are avoidable [4].

IVF is one of the most widely known techniques that assist infertility problems. Transvaginal oocyte retrieval is an ultrasound-guided procedure in which oocytes are aspirated using a needle connected to a suction pump. Several observational studies evaluated the complications associated with this procedure and reported very low rates of serious complications. However, the risks associated with oocyte retrieval should not be underestimated, as some rare complications may be life-threatening. The reported clinical complications after the procedure include vaginal and peritoneal bleeding, infection, urinary tract injury, and pseudoaneurysm. Rarely, ovarian bleeding may lead to severe hemoperitoneum, and the symptoms may appear either early or late (i.e., up to 28 hours) after the oocyte retrieval procedure [7-8]. Minor arterial/venous bleeding is considered a common complication, occurring in 1.4% to 18.4% of cases and is thought to arise secondary to direct trauma to the adjacent vessels. This is usually managed with local treatment such as applying local pressure [9].

One study reported that risk factors for severe intraperitoneal bleeding were low body mass index, a history of surgery, younger age, and a moderate ovarian response [10]. A pseudoaneurysm following transvaginal oocyte retrieval is an infrequent but potentially fatal complication; it is usually iatrogenic, caused by the trauma of the aspiration needle during the procedure. Two cases were reported with similar circumstances. The first case was a 34-year-old woman with primary infertility for eight years who presented with massive hematuria and hemodynamic instability following oocyte retrieval; she underwent an emergency cystoscopy under general anesthesia. Her care team noted that her bladder was filled with clots. Additionally, her care team reported a pseudoaneurysm near the right ureteric orifice, which was spurting blood [8]. The second case report was of a 36-year-old woman with secondary infertility who received routine transvaginal oocyte retrieval as part of her IVF treatment. Four days later, she presented with life-threatening hemorrhagic shock. Consequently, she underwent surgical laparotomy followed by CT and selective angiography, which demonstrated hemorrhage from a pseudoaneurysm of the obturator artery [9]. Both cases required life-saving blood transfusion and resuscitation.

The management of pelvic pseudoaneurysm depends on the stability of the patient’s clinical state for either open surgical repair or endovascular intervention. In hemodynamically stable patients, the treatment of choice is uterine artery embolization performed by an interventional radiologist. However, unstable patients should undergo laparotomy to discover the source of bleeding and treat it in the operating room [11]. Another case report of a 37-year-old woman who presented with painless vaginal bleeding and pulsating sensation at 12 weeks of gestation with the assistance of IVF was diagnosed as incomplete miscarriage. A magnetic resonance angiogram revealed a pelvic pseudoaneurysm. Thus, a selective angioembolization was performed successfully, and the patient improved clinically and radiologically; the pseudoaneurysm decreased in size and remained occluded. Her care team suspected the pseudoaneurysm was a complication of oocyte retrieval [12].

## Conclusions

We presented a rare case of uterine artery aneurysm and pseudoaneurysm due to oocyte retrieval for IVF. In the diagnosis process, a FAST scan was conducted first, followed by a CT scan that revealed active extravasation adjacent to the hyper-stimulated left ovary and severe hemoperitoneum. The patient was evaluated daily clinically and via laboratory investigations, and once she improved, she was discharged home. As this case highlights, the management of pseudoaneurysm depends mainly on patient stability.

## References

[1] Dar OH, Dar MA, Wagay MI, Hassan S, Qadir S (2016). Uterine artery pseudo-aneurysm: review of literature. West Afr J Radiol.

[2] Gürses C, Yilmaz S, Biyikli S, Yildiz IO, Sindel T (2008). Uterine artery pseudoaneurysm: unusual cause of delayed postpartum hemorrhage. J Clin Ultrasound.

[3] Jennings L, Presley B, Krywko D (2019). Uterine artery pseudoaneurysm: a life-threatening cause of vaginal bleeding in the emergency department. J Emerg Med.

[4] Katole A, Saoji AV (2019). Prevalence of primary infertility and its associated risk factors in urban population of central India: a community-based cross-sectional study. Indian J Community Med.

[5] Mohammad K, Ardalan A (2009). An overview of the epidemiology of primary infertility in Iran. J Reprod Infertil.

[6] Al-Turki HA (2015). Prevalence of primary and secondary infertility from tertiary center in eastern Saudi Arabia. Middle East Fertil Soc J.

[7] Levi-Setti PE, Cirillo F, Scolaro V (2018). Appraisal of clinical complications after 23,827 oocyte retrievals in a large assisted reproductive technology program. Fertil Steril.

[8] Jayakrishnan K, Raman VK, Vijayalakshmi VK, Baheti S, Nambiar D (2011). Massive hematuria with hemodynamic instability—complication of oocyte retrieval. Fertil Steril.

[9] Bolster F, Mocanu E, Geoghegan T, Lawler L (2014). Transvaginal oocyte retrieval complicated by life-threatening obturator artery haemorrhage and managed by a vessel-preserving technique. Ulster Med J.

[10] Zhen X, Qiao J, Ma C, Fan Y, Liu P (2010). Intraperitoneal bleeding following transvaginal oocyte retrieval. Int J Gynaecol Obstet.

[11] Belfort MA (2021). Postpartum hemorrhage: management approaches requiring laparotomy. UpToDate.

[12] Pappin C, Plant G (2006). A pelvic pseudoaneurysm (a rare complication of oocyte retrieval for IVF) treated by arterial embolization. Hum Fertil (Camb).

